# Draft Genome Sequences of Four Multidrug-Resistant Pseudomonas aeruginosa Isolates from Hospital Wastewater in Singapore

**DOI:** 10.1128/MRA.01193-18

**Published:** 2018-11-15

**Authors:** Charmaine Ng, Xiaoqiong Gu, Shin Giek Goh, Hongjie Chen, Laurence Haller, Boonfei Tan, Karina Yew-Hoong Gin

**Affiliations:** a Department of Surgery, National University of Singapore, Singapore, Singapore; b Department of Civil and Environmental Engineering, National University of Singapore, Singapore, Singapore; c Department of Biological Sciences, University of Alberta, Edmonton, Alberta, Canada; d NUS Environmental Research Institute (NERI), Singapore, Singapore; University of Maryland School of Medicine

## Abstract

Four multidrug-resistant Pseudomonas aeruginosa isolates were cultured from intensive care unit wastewater. All isolates exhibited resistance to carbapenem and extended-spectrum beta-lactam antibiotics. Genome characterization revealed the presence of beta-lactamase resistance genes (*bla*_PAO_ and *bla*_OXA_), and three out of the four isolates carried the *bla*_NDM-1_ gene encoding resistance against carbapenems.

## Abstract (precis)

Four multidrug-resistant Pseudomonas aeruginosa isolates were cultured from intensive care unit wastewater. All isolates exhibited resistance to carbapenem and extended-spectrum beta-lactam antibiotics.

## ANNOUNCEMENT

Clinical wastewater is a source of pharmaceutical by-products, pathogens, and antibiotic-resistant bacteria (1–3), and may facilitate the spread of antimicrobial resistance in receiving waters if not treated. Four multidrug-resistant Pseudomonas aeruginosa isolates (WPB098, WPB099, WPB100, and WPB101) were cultured from wastewater onto selective chromogenic media, CHROMagar Klebsiella pneumoniae carbapenemase (KPC) agar (CHROMagar, Paris, France) from hospital wastewater samples of a clinical isolation ward in Singapore as described in Haller et al. (2017) (2). Antimicrobial susceptibility profiles of the isolates were evaluated using the AST-GN79 cards on the Vitek 2 compact system (bioMérieux, Marcy L'Étoile, France) according to the manufacturer’s instructions. All isolates showed resistance to 16 of the antibiotics tested, amikacin (AMK), ampicillin (AMP), ampicillin-sulbactam (SAM), cefazolin (CFZ), cefepime (FEP), cefoxitin (FOX), ceftazidime (CAZ), ceftriaxone (CRO), ciprofloxacin (CIP), ertapenem (ETP), meropenem (MEM), gentamicin (GEN), nitrofurantoin (NIT), piperacillin-tazobactam (TZP), tobramycin (TOB), and trimethoprim-sulfamethoxazole (SXT). These results indicated that the isolated strains exhibit resistance to carbapenems and extended-spectrum beta-lactamase (ESBL) antibiotics.

For whole-genome sequencing, single colonies were picked from overnight growth and cultured in Luria Bertani broth overnight at 37°C. Total genomic DNA was extracted using the UltraClean microbial DNA isolation kit (Mo Bio Laboratories, Inc., Carlsbad, CA). Using the standard protocol, indexed sequencing libraries were prepared using a New England Biolabs Next Ultra DNA library prep kit for Illumina (New England BioLabs [NEB], Inc., MA, USA) and sequenced as paired-end reads (2 × 151 bp) using the Illumina HiSeq 4000 platform. A total of 26.1 million paired-end reads were obtained from four samples. Quality controlled reads with Phred scores of >20 were retained and assembled *de novo* using VelvetOptimiser 2.2.4 (4) with a minimum contig cutoff of 500 bp. Scaffolding was performed using Opera 1.4.1 (5) and finished using FinIS 0.3 with default settings (6). The genomes ranged between 6.5 and 6.9 Mbp and contained 80 to 98 contigs, with a G+C content of 66%. The assembled genomes had *N*_50_ values of between 234,330 and 312,695.

To identify antibiotic-resistant genes (ARGs) carried by each P. aeruginosa isolate, the assembled genomes were searched against the Resfinder 3.0 database using default parameters (7). All 4 isolates had ARGs that confer resistance to aminoglycoside [*aph*(*3*′)-*IIb*], beta-lactam (*bla*_PAO_ and *bla*_OXA_), fosfomycin (*fosA*), and phenicol (*catB7*) antibiotics, while isolates WPB099, WPB100, and WPB101 encoded additional ARGs resistant to fluoroquinolone (*crpP* and *qnrVC1*), macrolide-lincosamide-streptogramin B (*msrE*), sulfonamide (*sul2*), and trimethoprim (*dfrB5*) antibiotics. The *bla*_NDM-1_ gene, which encodes the New Delhi metallo-beta-lactamase enzyme capable of hydrolyzing ESBL and carbapenem antibiotics, was detected in genomes of isolates WPB099, WPB100, and WPB101.

### Data availability.

Whole-genome sequences of P. aeruginosa strains WPB098 to WPB101 were deposited in GenBank under accession numbers CP031876 to CP031879 (BioProject number PRJNA486613). Raw reads can be found in the NCBI Sequence Read Archive (SRA) under accession numbers SRR8002634 to SRR8002637.
